# Inhibition of the PI3K/AKT pathway potentiates cytotoxicity of EGFR kinase inhibitors in triple-negative breast cancer cells

**DOI:** 10.1111/jcmm.12046

**Published:** 2013-04-20

**Authors:** Yong Weon Yi, Wooyoung Hong, Hyo Jin Kang, Hee Jeong Kim, Wenjing Zhao, Antai Wang, Yeon-Sun Seong, Insoo Bae

**Affiliations:** a Department of Oncology, Lombardi Comprehensive Cancer Center, Georgetown UniversityWashington, DC, USA; b Department of Radiation Medicine, Lombardi Comprehensive Cancer Center, Georgetown UniversityWashington, DC, USA; c Department of Nanobiomedical Science and WCU (World Class University) Research Center of Nanobiomedical Science, Dankook UniversityCheonan, Korea; d Department of Biostatistics, Columbia UniversityNew York, NY, USA; e Herbert Irving Comprehensive Cancer Center, Columbia UniversityNew York, NY, USA

**Keywords:** triple-negative breast cancers, EGFR, PI3K/AKT, protein kinase inhibitors, cytotoxicity, synergism

## Abstract

Triple-negative breast cancers (TNBCs) are known to be intrinsically resistant to inhibitors for epidermal growth factor receptor (EGFR). Until now, clinical trials for TNBCs using EGFR inhibitors (EGFRis) as single agents have yielded disappointing results. Here, we report that combinatorial treatment using EGFRis, such as gefitinib or erlotinib, with PI3K/AKT pathway inhibitors (PI3K/AKTis) demonstrated a synergistic, anti-proliferative effect in cell lines of the basal-like (BL) subtype, a subtype of TNBC. Western blot analysis revealed that the gefitinib/PI-103 combination significantly reduced the level of both phospho-AKT and phospho-ERK in two susceptible BL subtype cell lines, SUM149PT and MDA-MB-468, whereas it had little or no effect on the level of phospho-ERK in two non-susceptible cell lines (HS578T and MDA-MB-231) of mesenchymal stem-like (MSL) TNBC subtype. The gefitinib/PI-103 combination also significantly induced caspase-3/7-mediated PARP cleavage and reduced two anti-apoptotic proteins, XIAP and Bcl-2 in the susceptible cell lines. In addition, the level of myeloid cell leukemia 1 (Mcl-1) protein was markedly decreased by gefitinib/PI-103 combination in the BL TNBC cells, but showed no significant change by this combination in MSL subtype cells. These results suggest that pharmacological inhibition of EGFR used in combination of PI3K/AKTis is a potential therapeutic approach to treat a subtype of TNBCs.

## Introduction

Triple-negative breast cancers (TNBCs) constitute up to 20% of all breast cancers and are characterized by a lack of oestrogen receptor (ER) and progesterone receptor (PR), as well as human epidermal growth factor receptor 2 (HER2) amplification 1. Although TNBCs have higher response rates to neoadjuvant chemotherapy, TNBC patients show a higher rate of recurrence and poorer prognosis than other types of breast cancers. Unfortunately, recent efforts on developing new target-based therapeutics in breast cancers have mostly shown little efficacy in clinical trials 2. These negative results are mainly owing to limited knowledge on the complexity and heterogeneity of breast cancers 3. Currently, no successful therapeutic target is known for TNBC treatment 1. Given the growing number of target-based agents, however, it is likely that studies will yield a more detailed understanding of molecular mechanisms and pathways that are important to carcinogenesis and/or cellular proliferation. It is further expected that breast cancer progression can be controlled by combining several compounds targeting different signalling pathways. In fact, recent reports suggest that TNBC is a group of heterogeneous cancers consisting of at least six subtypes based on gene expression profiles 4 and distinct protein kinases are specifically activated in different subtypes of breast cancer cells in clinical samples 5.

Epidermal growth factor receptor (EGFR) is a member of the type I transmembrane receptor tyrosine kinases (RTKs) of the ERBB/HER family, which includes ERBB2/HER2, ERBB3 and ERBB4 6, 7. Like many other RTKs, EGFR has important roles in proliferation and differentiation of normal cells and malignant transformation 6, 7. It has been well-established that EGFR is a major oncogenic factor and a promising therapeutic target in certain types of cancers, and many EGFR inhibitors (EGFRis), including monoclonal antibodies and small-molecule tyrosine kinase inhibitors, have been approved by the FDA for the treatment of several human cancers 6, 8. However, most studies on EGFR therapeutic potential have been focused in glioblastoma, lung cancer and head and neck cancers 9. Although EGFR is highly expressed in more than 50% of TNBCs, its role and therapeutic potential in breast cancers is poorly understood 2, 7, 9. Most breast cancer clinical trials with EGFRis as single agents have turned out to be disappointing 2, 9. Currently, only lapatinib, which inhibits both EGFR and ERBB2/HER2, in combination with capecitabine, has been approved by the FDA to treat patients with advanced or HER2-overexpressing metastatic breast cancer 2.

Here, we report that *in vitro* co-treatment of EGFRis and the phosphoinositide 3-kinase (PI3K)/AKT pathway inhibitors (PI3K/AKTis) enhances the anti-proliferative effects of EGFRis in two susceptible cell lines (SUM149PT and MDA-MB-468) which belong to the basal-like (BL) subtype of TNBC. Combinatorial treatment of gefitinib and PI-103 synergistically reduces both phospho-AKT and phospho-ERK in these cells. In addition, significant increase in apoptotic cell death is induced by the gefitinib/PI-103 combination in the BL subtype cell lines of TNBC.

## Materials and methods

### Cell culture and reagents

All cell lines, except for SUM149PT, were purchased from American Type Culture Collection (Manassas, VA, USA). MCF7 and MDA-MB-231 were maintained in Dulbecco's Modified Eagle Medium (DMEM) containing 5% heat inactivated fetal bovine serum (HI-FBS; HyClone, Logan, UT, USA) and 100 units/ml penicillin/streptomycin. HS578T, MDA-MB-468 and MDA-MB-436 were maintained in DMEM containing 10% HI-FBS and 100 units/ml penicillin/streptomycin. SUM149PT was maintained according to manufacturer's recommendations (Asterand, Detroit, MI, USA). The viability of cultured cells was monitored by the trypan blue dye exclusion test using the Luna Automated Cell Counter (Logos Biosystems, Gyunggi-Do, Korea). Cell culture reagents were purchased from Invitrogen (Carlsbad, CA, USA), Lonza (Basel, Switzerland) or Cellgro (Manassas, VA, USA). Protein kinase inhibitors were purchased from the following sources: BMS-599626, PI-103, PIK-90 and MK-2206 from Selleck Chemicals (Houston, TX, USA); erlotinib from LKT Laboratories (St. Paul, MN, USA); gefitinib from LC Labs (Woburn, MA, USA); PD-153035 from Calbiochem (Gibbstown, NJ, USA). Stock solutions of compounds were made with appropriate concentrations in dimethyl sulfoxide (DMSO) and stored at −20°C in small aliquots.

### MTT (3-(4,5-Dimethylthiazol-2-yl)-2,5-diphenyltetrazolium bromide) assays

Cell proliferation was assayed at ∼72 hrs after treatment of compounds by MTT assay as described previously 10, 11. In brief, cells were subcultured into 96-well plates according to their growth properties. About 72 hrs after treatment with compounds, viable cells were stained by adding 20 μl of 5 mg/ml MTT solution per 100 μl of growth medium. After incubating for 2–4 hrs at 37°C, the media were removed and 150 μl/well of absolute DMSO was added to dissolve the formazan. The absorbance of each well was measured by the ELx808 microplate reader (BioTek, Winooski, VT, USA) and viable cells are presented as a per cent of the control, untreated cells. The combination index (CI) 12 was calculated by CompuSyn software V1.0 (ComboSyn, Paramus, NJ, USA).

### Western blots and antibodies

Cells were lysed by cell lysis buffer [20 mM Tris-Cl (pH 8.0); 0.5 M NaCl; 0.25% Triton X-100; 1 mM EDTA; 1 mM EGTA; 10 mM β-glycerophosphate; 10 mM NaF; 300 μM Na_3_VO_4_; 1 mM benzamidine; 1 mM DTT; and 2 μM PMSF] and western blot and densitometric analyses were performed as described previously 10, 13. Antibodies used in this study were as follows: Mcl-1 (sc-20679), phospho-ERK1/2 (Y204/Y187) (sc-7383), ERK1 (sc-94) and HSP90 (sc-7947) from Santa Cruz (Santa Cruz, CA, USA); EGFR (#4405), phospho-Akt (Ser473) (#9271), Akt (#9272) and XIAP (#2045) from Cell Signaling (Danvers, MA, USA); PARP (556494) and Bcl-2 (551107) from BD Biosciences (San Jose, CA, USA); and α-tubulin, β-actin and horseradish peroxidase-conjugated secondary antibodies from Sigma-Aldrich (St. Louis, MO, USA). The chemiluminescence reagent was purchased from Thermo Scientific (Rockford, IL, USA).

### Caspase-3/7 activity assay

Activity of caspase-3/7 was measured by the Caspase-Glo 3/7 Assay Kit from Promega (Madison, WI, USA) according to manufacturer's instructions 10. The day after subculture, cells were treated with either gefitinib or PI-103 individually, or in combination for 30 hrs. Both attached and suspended cells were harvested, and the cell lysates were used to measure caspase-3/7 activity. The luminescence from each assay was measured by the Wallac Victor^2^ multimodal microplate reader (Perkin-Elmer Life Sciences, Boston, MA, USA) at the Genomics and Epigenomics Shared Resource at Georgetown University Medical Center. Lysis buffer with substrate was used as the blank. Relative luminescence units were normalized by protein concentration and adjusted to the value from vehicle-treated cells.

### Detection of apoptotic cell death

Apoptotic cell death was detected by annexin V/propidium iodide (PI) staining. Cells were incubated with either gefitinib or PI-103 individually, or in combination for 24 hrs. Both attached and floating cells were harvested and washed twice with cold DPBS. Flow cytometric analysis with annexin V/PI staining was performed at the Flow Cytometry and Cell Sorting Shared Resource at Georgetown University Medical Center.

### Statistical analysis

Two-sample *t*-tests with unequal variances were conducted for comparisons in Figure 2; analysis of variance (anova) and subsequent two-sample *t*-tests using Tukey's multiple comparison adjustments for comparisons in Figures 3 and 5. All statistical tests were two-tailed and employed at a significance level of 5% to determine whether a significant difference exists in the EGFRis levels between two experiments. Data were analysed using SAS version 9.2. * indicates *P* < 0.05; ** indicates *P* < 0.01 and *** indicates *P* < 0.001.

## Results

### Overexpression of EGFR in TNBC cell lines

**Fig. 1:**
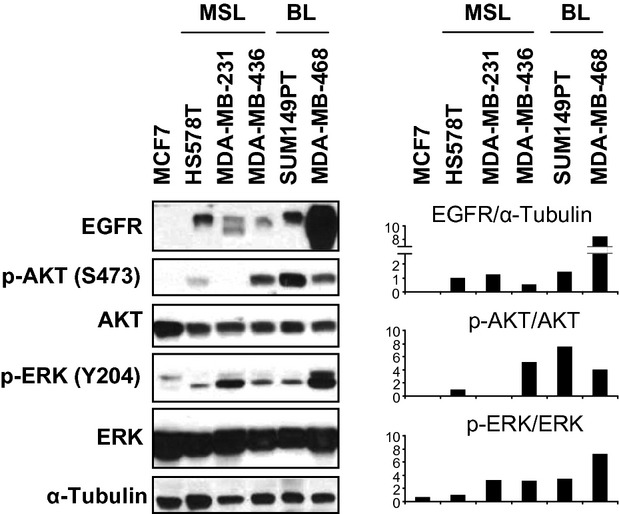
EGFR is overexpressed in TNBC cell lines. Cells were harvested the day after subculture and cell lysates were analysed by western blot with the indicated antibodies. α-tubulin was used as a loading control. MSL: mesenchymal stem-like; BL: basal-like.

As previously mentioned, EGFR is known to be overexpressed in more than 50% of TNBCs 2, 7, 9. To determine whether the level of EGFR is high in TNBC cell line models, we performed western blot analysis with 5 TNBC cell lines. As shown in Figure 1, all of the TNBC cell lines examined expressed a high level of EGFR compared with the luminal breast cancer cell line, MCF7. Among these cell lines, the level of EGFR protein was low in MDA-MB-436 cells, whereas the highest expression of EGFR was observed in MDA-MB-468 cells. Interestingly, the level of phospho-AKT (S473) was significantly high in 3 of 5 TNBC cell lines. As previously reported 10, breast cancer cell lines carrying BRCA1 mutations, have relatively high level of phospho-AKT (S473). In addition, significant levels of phospho-ERK (especially phospho-ERK2) were detected in all TNBC cell lines. Two cell lines, which can be subgrouped as the BL subtype of TNBC 4, expressed high levels of EGFR and phospho-AKT (S473), whereas the levels of phospho-AKT (S473) were varied in all three cell lines of the mesenchymal stem-like (MSL) subtype. Because AKT is known as a central converging node for many oncogenic upstream kinases 14 and confers resistance to many cancer therapeutics 15, we determined the effects of combining PI-103, a PI3K/AKTi, with several EGFRis in TNBC cell lines.

### Combinatorial treatment with PI3K/AKT pathway inhibitors enhances cytotoxic effects of EGFR inhibitors in cell lines of the BL subtype

**Fig. 2:**
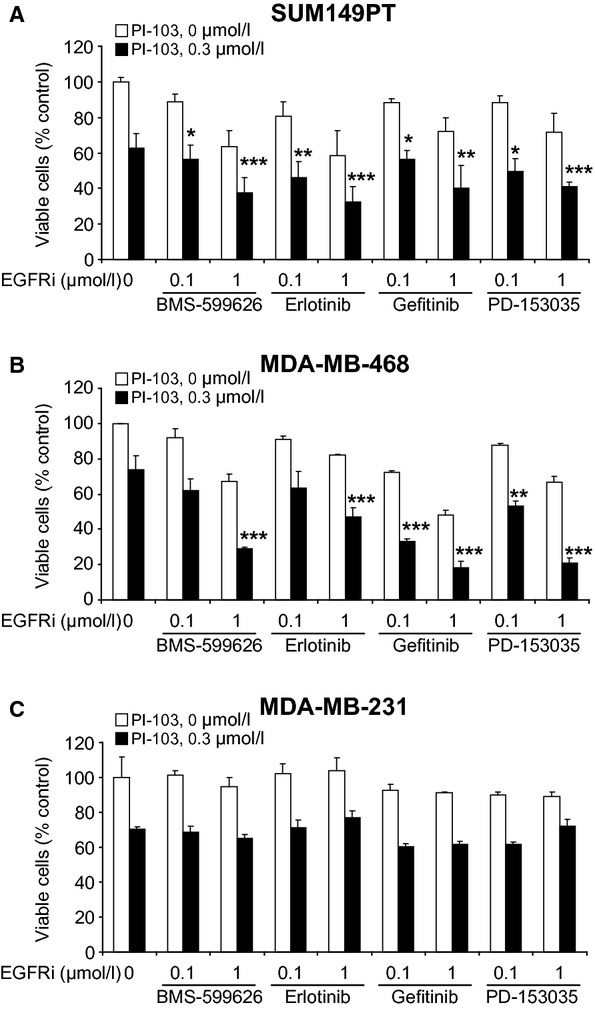
Co-treatments of PI-103 and EGFR inhibitors enhance cytotoxicity in SUM149PT (**A**) and MDA-MB-468 (**B**) but not MDA-MB-231 (**C**) cells. Cells were treated with 0.3 μM of PI-103 in combination with different concentrations (0.1 and 1 μM) of EGFR inhibitors for ∼72 hrs. Cell viability was measured by MTT assay as described in Materials and methods. Data from two independent experiments performed in triplicate are shown as mean ± SEM. *P < 0.05; **P < 0.01; ***P < 0.001.

To determine any combinatorial effects of PI-103 and EGFRis (Table 1), we chose three distinct cell lines, SUM149PT, MDA-MB-468 and MDA-MB-231, that expressed high level of EGFR. The level of phospho-AKT (S473), however, was much lower in MDA-MB-231 cells than in SUM149PT and MDA-MB-468 cells (Fig. 1). These cells were treated with PI-103 in combination with several EGFRis for up to 72 hrs and the viable cells were measured by MTT assay. Although the level of phospho-AKT (S473) was quite different in these cell lines, treatment of PI-103 alone inhibited the proliferation of three cell lines to similar degree (Fig. 2). Most of the EGFRis, as single agents, showed little or no effect on the proliferation of MDA-MB-231 cells up to 1 μM (Fig. 2C). On the contrary, EGFRis inhibited the proliferation of SUM149PT and MDA-MB-468 cells in a dose-dependent manner (Fig. 2A and B). In addition, combinatorial treatments of these EGFRis with 0.3 μM of PI-103 further reduced the proliferation of SUM149PT and MDA-MB-468 cells, whereas no significant enhancement of anti-proliferation by these combinations was observed in MDA-MB-231 cells.

**Table 1:** Protein kinase inhibitors and their known biochemical IC_50_ used in this study

Inhibitor	Known targets (IC_50_, nM)	Reference
Gefitinib	EGFR (3), HER2 (1830)	16, 17
Erlotinib	EGFR (2), HER2 (350), KDR (600)	16
PD-053035	EGFR (0.025)	18
BMS-599626	EGFR (22), HER2 (32), HER4 (190)	19
PI-103	DNA-PK (2), PI3Kα (8), mTORC1 (20), PI3Kδ (48), PI3Kβ(88), PI3Kγ (150), mTORC2 (83)	20
PIK-90	PI3Kα (11), DNA-PK (13), PI3Kγ (18), PI3KC2a (47), PI3Kδ (58), PI3KC2b (64), PI3Kβ(350)	20
MK-2206	AKT1 (5), AKT2 (12), AKT3 (65)	21

**Fig. 3:**
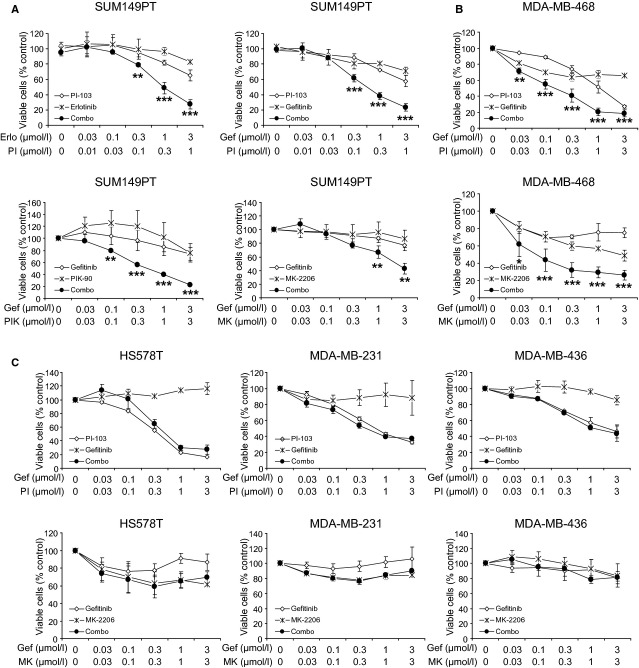
Combinations of PI3K/AKT inhibitors and EGFR inhibitors exert synergistic cytotoxicity in the susceptible cells. (**A**) SUM149PT cells were treated with various combinations of PI3K/AKT inhibitors and EGFR inhibitors for ∼72 hrs. (**B**) MDA-MB-468 cells were treated with gefitinib in combination with either PI-103 or MK-2206 for ∼72 hrs. (**C**) The non-susceptible cells (HS578T, MDA-MB-231, MDA-MB-436) were treated with gefitinib in combination with either PI-103 or MK-2206 for ∼72 hrs. (A–C) Viable cells were measured by MTT assay as described in Materials and methods. Data from two independent experiments performed in triplicate are shown as mean ± SEM. Abbreviations: Erlo, erlotinib; Gef, gefitinib; PI, PI-103; MK, MK-2206. *P < 0.05; **P < 0.01; ***P < 0.001.

To further explore these observations, we conducted a series of MTT assays with expanded cell lines. Cells were treated with increasing amounts of compounds either as single agents or in combination with fixed ratio up to 72 hrs, and cell viability was measured by MTT assay. Combinatorial treatment of PI-103 with two clinical EGFRis, erlotinib or gefitinib, synergistically inhibited the proliferation of SUM149PT cells with CI_50_ of 0.382 and 0.178 respectively (Fig. 3A upper panel). Synergistic effects of the gefitinib/PI-103 combination were also confirmed in MDA-MB-468, another BL subtype cell line (Fig. 3B upper panel). These synergistic effects were not observed in three cell lines (HS578T, MDA-MB-231 and MDA-MB-436) of the MSL subtype (Fig. 3C upper panel). In addition, co-treatment of gefitinib with either PIK-90 (a specific inhibitor of PI3Kα and DNA-PK) or MK-2206 (an allosteric inhibitor of AKT; Table 1) synergistically inhibited the proliferation of SUM149PT cells (Fig. 3A lower panel). Combined treatment of gefitinib/MK-2206 also enhanced the anti-proliferative effects in MDA-MB-468 cells (Fig. 3B lower panel). Again, no significant synergisms of the gefitinib/MK-2206 combination were observed in three cell lines of the MSL subtype (Fig. 3C lower panel). All these results suggest that PI3K/AKTis potentiate the anti-proliferative ability of EGFRis in the BL subtype TNBC cell lines.

### Combination of gefitinib with PI-103 synergistically inhibits both AKT and ERK pathways in the susceptible cell lines

**Fig. 4:**
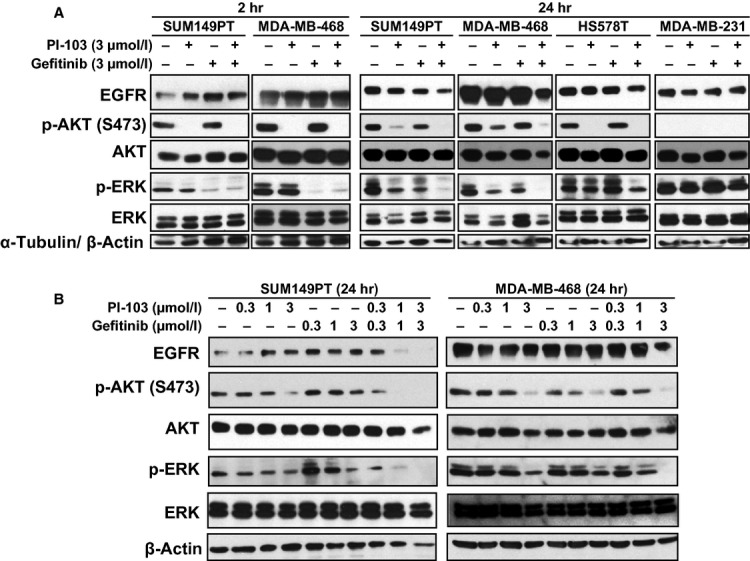
Combination of PI-103 with gefitinib abolishes AKT and ERK pathways in the susceptible cells. (**A**) Cells were treated with either gefitinib (3 μM), PI-103 (3 μM) alone or a combination of both drugs for 2 or 24 hrs. α-tubulin (for 2-hr treatment) or β-actin (for 24-hr treatment) was used as a loading control. (**B**) Two susceptible cell lines (SUM149PT and MDA-MB-468) were treated with increasing amounts of gefitinib, PI-103 or in combination of both drugs for 24 hrs. β-actin was used as a loading control. (A–B) Western blot analysis was performed with the indicated antibodies. Representative data from two independent experiments are shown.

We examined effects of the gefitinib/PI-103 combination on signalling pathways by a series of western blot analyses. Short-term treatment (2 hrs) of PI-103, as a single agent, significantly reduced phospho-AKT (S473) levels in all four cell lines tested (Fig. 4A and data not shown). However, short-term treatment of gefitinib, as a single agent, significantly reduced the level of phospho-ERK only in two susceptible cell lines, SUM149PT and MDA-MB-468 (Fig. 4A and data not shown). Short-term co-treatment of both drugs showed similar effects on the levels of phospho-AKT and phospho-ERK compared with the treatment of both drugs as single agents in the susceptible cells. Interestingly, long-term treatment (24 hrs) of either drug as a single agent resulted in partial restoration of either phospho-AKT or phospho-ERK in SUM149PT and MDA-MB-468 cell lines (Fig. 4A). Combinatorial treatment of gefitinib with PI-103, however, synergistically suppressed the restored level of phospho-AKT and phospho-ERK in these BL subtype cell lines. Little or no effects of this combination were observed in two non-susceptible cell lines. The effect of this combination on the levels of phosphorylation of AKT and ERK was dose dependent in both SUM149PT and MDA-MB-468 cells (Fig. 4B).

### Co-treatment of gefitinib and PI-103 induces caspase-dependent apoptosis in the susceptible cell lines

**Fig. 5:**
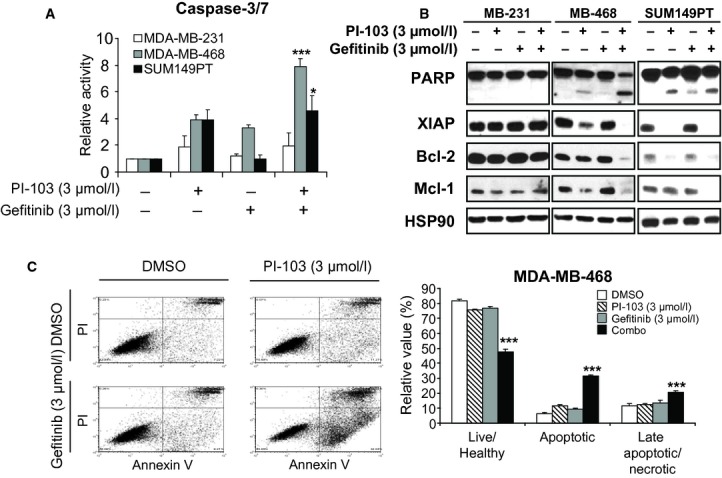
Combination of PI-103 with gefitinib synergistically induces apoptotic cell death in the susceptible cells. (**A**) Cells were treated with the indicated amounts of compounds for 30 hrs and caspase-3/7 activities were measured as described in Materials and methods. Data from three independent experiments are shown as mean ± SEM. *P < 0.05; ***P < 0.001. (**B**) MDA-MB-468 cells were treated as indicated and both attached and floating cells were harvested and stained with annexin V and PI. (Left) Representative flow cytograms are shown. (Right) Data are shown as mean ± SD performed in triplicate. ***P < 0.001. (**C**) Cells were treated with the indicated amounts of compounds for 30 hrs and cell lysates were analysed by western blot with the indicated antibodies. Representative data from three independent experiments are shown. HSP90 was used as a loading control.

To determine apoptotic cell death induced by the gefitinib/PI-103 combination, we measured caspase-3/7 activity in cells treated with either drug alone or in combination for 30 hrs. In MDA-MB-231 cells, marginal increases in caspase-3/7 activity were observed in either gefitinib or PI-103 treatment, but no significant synergism of both drugs was detected (Fig. 5A). By contrast, in two BL subtype cell lines, MDA-MB-468 and SUM149PT, treatment of gefitinib or PI-103 as single agents induced increased activity of caspase-3/7 and the combination of gefitinib/PI-103 markedly increased caspase-3/7 activity (Fig. 5A).

Apoptotic cell death was further confirmed by annexin V/PI staining followed by flow cytometric analysis in MDA-MB-468 cells treated with either drug alone or in combination of both drugs for 24 hrs. Treatment of PI-103 or gefitinib alone marginally induced early apoptotic cell death (Fig. 5B). When the cells were treated with combination of gefitinib/PI-103, early apoptotic cell death was evident as 5-fold increase compared with the vehicle-treated control. Combination treatment also increased the late apoptotic/necrotic cell death ∼2-fold over the control treatment.

Western blot analysis was performed to further analyse the synergistic effects of gefitinib/PI-103 in cell lysates treated with either drug as a single agent or a combination of both drugs for 30 hrs. In the two susceptible cell lines (MDA-MB-468 and SUM149PT), combination of gefitinib/PI-103 synergistically induced the level of PARP cleavage (Fig. 5C). In addition, the levels of three anti-apoptotic proteins, XIAP (X-linked inhibitor of apoptosis protein), Bcl-2 and Mcl-1, were profoundly reduced in cells treated with gefitinib/PI-103 combination than cells treated with either drug alone. Under this condition, no significant changes in the cleavage of PARP and the level of XIAP, Bcl-2 and Mcl-1 proteins were detected in MDA-MB-231 cells. All these results suggest that the combination of gefitinib/PI-103 synergistically induces apoptotic cell death in the BL subtype cell lines of TNBC.

## Discussion

Our present study demonstrates EGFR as a potential target by combining EGFRis with PI3K/AKTis in a subtype of TNBC. As shown in our present study and others 22, EGFR is highly expressed in TNBC, but the efficacy of clinical EGFRis, such as gefitinib and erlotinib, as single agents is limited in pre-clinical TNBC cell line models. Interestingly, we found that a high level of EGFR was observed with elevated levels of phospho-AKT in two BL subtype cell lines of TNBC and combined treatment with PI3K/AKTis significantly enhanced the anti-proliferative effects of EGFRis in these cell lines. By contrast, PI3K/AKTis did not synergize the effects of EGFRis in the MSL subtype cell lines with high levels of EGFR overexpression and heterogeneous level of phospho-AKT expression. Although the PI3K/AKT pathway is known to be downstream of EGFR activation 6–8, treatment of gefitinib did not affect phospho-AKT (S473) levels in all the cell lines tested in this study. This raises the possibility that the activation of the PI3K/AKT pathway might be affected by other upstream kinases such as IGF-1R or MET in cells resistant to EGFRis 8. Overexpression of MET has been found in tissues derived from breast cancer patients 23. Together with other receptor tyrosine kinases, such as EGFR and KIT, MET have been established as those of many distinctly regulated genes in the basal-like breast cancer 23, which shares many features with TNBC 24, 25. All TNBC cell lines used in this study expressed high levels of MET compared with the luminal cell line, MCF7 (data not shown). A recent study reported that the MET ligand, hepatocyte growth factor (HGF), from fibroblasts paracrinely mediated the resistance of TNBC cells to EGFRis 26. However, further investigation is needed to dissect the role of MET in the intrinsic resistance to EGFRis and differential responses to the combination of PI3K/AKTis and EGFRis in TNBC cell lines.

In the present study, 24-hr treatment of PI-103 or gefitinib as single agents resulted in partial restoration of either phospho-AKT or phospho-ERK in two susceptible BL subtype cell lines (SUM149PT and MDA-MB-468), while 2-hr treatment with these drugs completely inhibited phospho-AKT and phospho-ERK, respectively. More interestingly, the combination of gefitinib/PI-103 synergistically reduced the restored levels of phospho-AKT and phospho-ERK in these cell lines. However, gefitinib, either as a single agent or in combination with PI-103, did not significantly inhibit the levels of phospho-ERK in the non-susceptible MSL cell lines (HS578T and MDA-MB-231). Unfortunately, the underlying molecular mechanism of this difference (susceptible cells *versus* non-susceptible cells) is not yet understood. Mutations of KRAS, an upstream activator of the MEK/ERK pathway, have known to be associated with primary resistance to both gefitinib and erlotinib in lung adenocarcinoma 27. However, association of KRAS mutations with resistance to anti-EGFR therapies has not been established in breast cancers. Further studies will be required to determine the upstream factor(s), which confer resistance to the gefitinib-mediated suppression of phospho-ERK in the MSL subtype of TNBC cells.

Similar to our results in the susceptible cells, reactivation of PI3K and ERK pathway through activation of upstream RTKs has recently been described in several cancer cell lines when treated with PI3K and/or mTOR inhibitors 28–30, 31. The efficacy of single agents might be limited because the inhibition of PI3K/AKT/mTOR pathway relieves the negative feedback loops that leads to reactivation of upstream RTKs such as HER2 and HER3 28–31. Our data suggest that the EGFR might contribute reactivation of either AKT or ERK pathway and combined inhibition of PI3K/AKT and EGFR/ERK pathway could provide an efficient strategy to inhibit the proliferation of susceptible TNBC cells. Further study with expanded TNBC cell lines needs to identify the subset(s) of TNBC cells that respond to EGFRi/PI3Ki combination and to dissect more detailed molecular mechanism that cause the reactivation of either ERK or PI3K pathway by administration of single agents in the susceptible cells.

In this study, we demonstrated that the combination of gefitinib/PI-103 synergistically changed apoptotic markers, namely induction of PARP cleavage and reduction of anti-apoptotic XIAP and Bcl-2 proteins in two susceptible cell lines, whereas no significant changes were observed in non-susceptible MDA-MB-231 cells. Regarding this, XIAP has been reported to confer acquired resistance to GW583340 (an EGFR/HER2 inhibitor) in inflammatory breast cancer cell lines, SUM190 and SUM149, and down-regulation of XIAP by a small-molecule inhibitor, embelin, which inhibits the XIAP/pro-caspase-9 interaction, decreases viability of these cells 32.

We also found that Mcl-1 was synergistically reduced by the gefitinib/PI-103 combination in SUM149PT and MDA-MB-468 cells. Mcl-1 is a major member of anti-apoptotic Bcl-2 family 33. Overexpression of Mcl-1 is associated with poor prognosis in many types of cancers including breast cancers 34. It has been proposed that increased levels of Mcl-1 are due to its stabilization by altered post-translational ubiquitination 34. In fact, mTORC1, a downstream target of AKT, can promote survival of the murine lymphoma model by stabilizing Mcl-1 35. Stability of Mcl-1 is also negatively regulated by phosphorylation at its Ser159 residue, which is mediated by GSK3β 36. Phosphorylation of GSK3β at Ser9 by AKT has been reported to inhibit its activity 37, 38. Consistently, treatment of PI-103 alone or in combination of gefitinib/PI-103 reduced the levels of Mcl-1 in two susceptible cell lines. Taken together, these results suggest that the EGFR/ERK pathway has a potential role in the regulation of Mcl-1 protein levels in cancer cells of the BL subtype of TNBC.

There is an urgent need for effective therapeutic regimens to treat TNBCs because these tumours constitute up to 20% of newly diagnosed breast cancers, more frequently affect young women and women of African ancestry and are biologically more aggressive and deadly 4, 9. Fortunately, recent advances in molecular profiling provide insights into the heterogeneity and subgroups of TNBCs 39. Given these advances, studying drug responses in specific subtypes of TNBCs will expand our understanding of these tumours for more effective therapies.
